# Multimeric CAX complexes and Ca^2+^ signaling – beyond humdrum housekeeping

**DOI:** 10.1093/jxb/erx227

**Published:** 2017-09-05

**Authors:** Yi Ma, Gerald A Berkowitz

**Affiliations:** Agricultural Biotechnology Laboratory, Storrs Rd, Unit, Department of Plant Science and Landscape Architecture, University of Connecticut, Storrs, CT,USA

**Keywords:** Ca^2+^ signaling, calcium, CAX, guard cells, homeostasis, mesophyll, protein interaction, signaling, transport

## Abstract

This article comments on:

Hocking B, Conn SJ, Manohar M, Xu B, Athman A, Stancombe MA, Webb AR, Hirschi KD, Gilliham M. 2017. Heterodimerization of Arabidopsis calcium/proton exchangers contributes to regulation of guard cell dynamics and plant defense responses. Journal Experimental Botany **68**, 4171–4183.

**Vacuolar Ca^2+^/H^+^antiporters (CAXs) contribute to Ca^2+^homeostasis within plant cells. But do they do more? Arabidopsis CAX isoforms have heretofore not been known to act in concert, but Hocking *et al.* (2017) now present a plethora of evidence suggesting that this underlies plasticity in response to environmental cues. CAX1 and CAX3 may form heteromeric transporters that impact guard cell function/leaf gas exchange, and leaf (mesophyll) cell responses to biotic stress.**

Ca^2+^: ‘You can’t live without it, you can’t live with it’ (at too high levels). In fact, you can’t even die (i.e. through apoptosis) without it! Calcium is a ubiquitous signal within cells and its temporally transient elevation within the cytosol is a key activator of numerous signaling events in every known prokaryotic and eukaryotic cell – and it has probably been this way since the earliest cells evolved (Clapham, 2007). Alongside these cytosolic Ca^2+^ (Ca^2+^_cyt_) signaling ‘spikes’, cell functions are severely impaired at homeostatic Ca^2+^_cyt_ above about 100 to 200 nM. Thus, proper cell and organism function, growth, and development require molecular transport systems that maintain low basal Ca^2+^_cyt_ and also ‘sweep’ away elevated Ca^2+^_cyt_ after signaling events.

Tonoplast Ca^2+^/H^+^ antiporters (CAXs) contribute to this vital system by providing a transport pathway for Ca^2+^ movement from the cytosol to the vacuole lumen against an extreme concentration gradient. This is the paradigm encompassing much of what we currently know about tonoplast-localized CAXs such as CAX1 and CAX3. In fact, we refer here to CAXs as having a ‘sweeping’ function to highlight what could be called a ‘housekeeping’ activity. This term, housekeeping, was also used with reference to CAXs by one of the authors of Hocking *et al.* in a recent review of CAX involvement in transport and signaling events (Pittman and Hirschi, 2016). Perhaps, as shown in this most recent work by Hocking *et al*., altered expression of CAX isoforms as well as their heteromeric association in native protein complexes belie this notion of CAXs as ‘just’ attending to humdrum housekeeping chores. In presenting some new phenotypes of CAX mutants, and hinting at underlying mechanisms associated with these phenotypes, the authors break new ground about this important family of cation transporters in a number of ways. It may be that CAX proteins in vacuolar membranes have finer points of function other than rudimentary Ca^2+^_cyt_ homeostasis-inducing, clearing activities.

## CAX functional plasticity

One of the technical limitations of CAX research until now has been that in order to demonstrate their transport function (for example, upon expression in plant mutants lacking endogenous CAX genes or in heterologous systems), the CAX polypeptide had to be expressed as a truncated protein variant. The CAX N-terminal regulatory region has autoinhibitory activity. Hence, studies were done on truncated translation products of ‘sCAX’ coding sequences (Manohar *et al.*, 2011). Work in Hocking *et al*. may portend new advancements because coexpression (in yeast mutants) of full-length CAX1 and CAX3 resulted in the generation of a functional transporter. Importantly, this CAX transporter had different biochemical properties than that displayed by CAX1 alone (expressed as the truncated ‘sCAX1’ polypeptide). Perhaps, then, native CAX exchangers comprising both CAX1 and CAX3 polypeptides might have altered function in native membranes as compared to dimer transporters made up solely of CAX1 coding sequences. This possibility underlies some of the newly developed conjectures of CAX functional plasticity presented in Hocking *et al*.

In conjunction with the transport and biochemical analyses of CAX heterodimers, Hocking *et al.* present some well-crafted studies of CAX expression patterns that suggest CAXs may function as heterodimers in the plant under various conditions. Laser capture microdissection combined with single-cell RNA analyses showed that although CAX1 predominates in vacuoles of leaf mesophyll cells, CAX3 is normally present along with CAX1 in guard cells. Further, they found that in leaf mesophyll cells, CAX3 transcription (and translation) is increased upon perception of the presence of pathogens. Bimolecular fluorescence complementation analysis of CAX1:CAX3 association documented that these CAX isoforms are capable of forming dimers (although whether this actually occurs in native membranes is unresolved).

These studies, approaching the question of whether CAX polypeptides are capable of, and do, function as heteromeric proteins *in situ* using different experimental approaches led the authors to speculate that CAX assembly as heteromeric dimers in the plant could provide some enhanced ability to respond to environmental perturbations. Using several experimental approaches, they surmised that during their protoplast preparation procedures, CAX3 protein generation increased over time. Viewing ‘protoplasting’ as a proxy for general stress responses, this led to speculation that CAX3 expression and, hence, CAX1/CAX3 dimer formation may provide plant cells with a tonoplast Ca^2+^/H^+^ antiporter with altered functional properties that provides benefits under a range of stress conditions. The authors noticed that some phenotypes displayed by *cax1/cax3* double mutants related to their presence in guard cells, and their function related to facilitating Ca^2+^ sequestration in the vacuole. A model was developed that linked CAX1–CAX3 function in the guard cell tonoplast to regulation of apoplastic Ca^2+^ and maintenance of normal stomatal aperture during changes in extracellular Ca^2+^.

## New possibilities

Hocking *et al*.’s biochemical analysis of the transport properties of CAX1–CAX3 dimers upon expression of the full-length coding sequences together in yeast mutants breaks new ground, and their work represents the first functional analysis of these transporters in the presence of their autoinhibitory domains. However, the technical challenge of characterizing the nature of a possibly heteromeric transport protein as it exists in native plant membranes precludes some definitive conclusions about the molecular basis for the mutant phenotypes. We can be certain from the work of these authors that CAX1 and CAX3 are capable of binding to themselves as well as each other. However, we do not know if native tonoplast membranes (in which both polypeptides are present) have CAX1 and CAX3 homodimers as well as the heterodimer. Future experiments involving immunoprecipitation of native CAX protein complexes and interrogation of the captured proteins with isoform-specific antibodies could resolve this particular issue.

Clearly, the work should be viewed in the context of the role CAX antiporters play in shaping plant cell responses to external signals that are mediated by Ca^2+^ acting as a cytosolic secondary messenger. Ca^2+^ signaling is of paramount importance to a myriad range of plant cell responses to environmental, developmental, and physiological cues. However, there is much still undefined at the molecular level regarding how Ca^2+^_cyt_ elevation acts as a secondary messenger to initiate a *specific* downstream response. The authors present their analyses of vacuolar Ca^2+^ sequestration facilitated by antiporters comprising CAX1 and CAX3 as a (perhaps) non-static responder to extracellular events leading to Ca^2+^_cyt_ signaling. They do not conceptualize their tonoplast Ca^2+^ sequestration system in the context of what Richard Tsien and colleagues (Wheeler *et al.*, 2012) conceive of as ‘private lines of communication’.

Could the CAX Ca^2+^ sequestration system be functionally linked to protein complexes that facilitate a specific Ca^2+^ signal transduction pathway (i.e. downstream from a specific external cue)?

An example of this point is as follows. Hocking *et al*. provide some intriguing evidence that CAX antiporters act in pathogen defense responses: application of flg22 (a peptide corresponding to a portion of the bacterial motor organ protein flagellin) stimulates CAX3 expression in mesophyll cells. Moreover, the flg22 peptide is recognized by a specific plasmalemma receptor, FLS2, and flg22 binding to its cognate receptor initiates an immune signaling cascade that requires Ca^2+^_cyt_ elevation (Chinchilla *et al.*, 2006). Early speculation that FLS2 acts in membrane microdomains that function as platforms for immune signaling (Qi and Katagiri, 2012) have recently been confirmed (Bücherl *et al.*, 2017). In this most recent paper, Bücherl *et al*. used single-particle tracking to suggest that specific plasmalemma receptors such as FLS2 that initiate different signaling pathways activated by Ca^2+^_cyt_ elevation exist as protein complexes associated with the proteins acting in the downstream steps of the individual signaling pathways.

Such protein complexes acting in Ca^2+^ signaling microdomains also underlie animal cell function. An archetypal example of such a Ca^2+^ signaling complex is the cell membrane Ca^2+^ channel Orai1 and the endoplasmic reticulum (ER)-localized Ca^2+^-binding protein STIM1. They functionally interact at physical junctures where the ER and cell membrane are linked in a multi-membrane signaling complex (Ambudkar *et al.*, 2017) and are involved in Ca^2+^ elevation in microdomains (Lee *et al.*, 2010). This Orai1:STIM1 ER:cell membrane signaling paradigm provides the basis for conjecture about similar Ca^2+^ signaling in plant cells.

Extending the points made by Hocking *et al*., we might further speculate that there are tonoplast:plasmalemma junctions that allow CAX complexes to respond to individual Ca^2+^ signaling events (such as flg22 binding to FLS2) occurring in local domains of the plasmalemma. CAX proteins are involved in numerous Ca^2+^ signaling cascades (e.g. the involvement of CAX3 in salinity responses; Manohar *et al.*, 2011). Thus, physical association of the tonoplast with the plasmalemma in microdomains could provide the private lines of communication envisioned by Wheeler *et al.* (2012) for CAX transporters to specifically ‘shape’ numerous Ca^2+^ signaling events in plants on a specific, individual basis. This conjecture is quite a bit down the line from the work shown in Hocking *et al.*, but their conclusion that CAX antiporters could have different functional properties depending on the specific isoforms making up the dimer protein does raise it as a possibility.

## References

[CIT0001] Ambudkar IS, de Souza LB, Ong HL. 2017 TRPC1, Orai1, and STIM1 in SOCE: Friends in tight spaces. Cell Calcium, doi: 10.1016/j.ceca.2016.12.009 10.1016/j.ceca.2016.12.009 PMC5466534 28089266

[CIT0002] Bücherl CA, Jarsch IK, Schudoma C, Segonzac C, Mbengue M, Robatzek S, MacLean D, Ott T, Zipfel C. 2017 Plant immune and growth receptors share common signalling components but localise to distinct plasma membrane nanodomains. eLife 6, e25114. 28262094 10.7554/eLife.25114 PMC5383397

[CIT0003] Chinchilla D, Bauer Z, Regenass M, Boller T, Felix G. 2006 The Arabidopsis receptor kinase FLS2 binds flg22 and determines the specificity of flagellin perception. The Plant Cell 18, 465–476. 16377758 10.1105/tpc.105.036574 PMC1356552

[CIT0004] Clapham DE. 2007 Calcium signaling. Cell 13, 1047–1058. 10.1016/j.cell.2007.11.028 18083096

[CIT0005] Hocking B, Conn SJ, Manohar M, Xu B, Athman A, Stancombe MA, Webb AR, Hirschi KD, Gilliham M. 2017 Heterodimerization of Arabidopsis calcium/proton exchangers contributes to regulation of guard cell dynamics and plant defense responses. Journal Experimental Botany 68, 4171–4183. 10.1093/jxb/erx209 PMC5853972 28645169

[CIT0006] Lee KP, Yuan JP, Hong JH, So I, Worley PF, Muallem S. 2010 An endoplasmic reticulum/plasma membrane junction: STIM1/Orai1/TRPCs. FEBS Letters 584, 2022–2077. 19944100 10.1016/j.febslet.2009.11.078 PMC2866752

[CIT0007] Manohar M, Shigaki T, Mei H, Park S, Marshall J, Aguilar J, Hirschi KD. 2011 Characterization of Arabidopsis Ca2+/H+ exchanger CAX3. Biochemistry 50, 6189–6195. 21657244 10.1021/bi2003839

[CIT0008] Pittman JK, Hirschi KD. 2016 CAX-ing a wide net: Cation/H^+^ transporters in metal remediation and abiotic stress signaling. Plant Biology 18, 741–749. 27061644 10.1111/plb.12460 PMC4982074

[CIT0009] Qi Y, Katagari F. 2012 Membrane microdomain may be a platform for immune signaling. Plant Signaling & Behavior 7, 454–456. 22499178 10.4161/psb.19398 PMC3419031

[CIT0010] Wheeler DG, Groth RD, Ma H, Barrett CF, Owen SF, Safa P, Tsien RW. 2012 CaV1 and CaV2 channels engage distinct modes of Ca2+ signaling to control CREB-dependent gene expression. Cell 149, 1112–1124. 22632974 10.1016/j.cell.2012.03.041 PMC3654514

